# Selection strategy and technical variations of extraanatomic bypass in surgical management of complex and recurrent aortic coarctation and hypoplastic aortic arch

**DOI:** 10.1186/1749-8090-8-S1-O16

**Published:** 2013-09-11

**Authors:** E Delmo Walter

**Affiliations:** 1 Cardiothoracic Surgery, Deutsches Herzzentrum Berlin, Berlin, Germany

## Objective

We report the operative selection strategy and technical variations of extraanatomic bypass to correct complex and recurrent coarctation of aorta and hypoplastic aortic arch, and their long-term outcome.

## Method

In 1989-2012, 60 patients (mean age 29±6.7years) with complex aortic coarctation (n=33), recurrent coarctation (n=27; anastomosis pseudoaneurysm in 10), underwent correction using extraanatomic bypass, either or without extracorporeal circulation. The decision to use extracorporeal circulation was based on the anatomical location of the coarctation, length of hypoplasia and history of previous repair. Various extraanatomic bypass strategies included left subclavian artery (LSCA) to descending aorta (DA) (n= 38), right subclavian artery (RSCA) to left carotid artery (LCA) (n=2), LCA to LSCA (n=3), LCA to DA (n=2), ascending aorta (AA) to LSCA (n=3), AA to DA (n=4), aortic arch to DA (n=3) and AA to abdominal aorta (n=5). We choose the size of the graft according to the diameter of the ascending aorta.. Preoperatively, mean systolic blood pressure was 130 ±30mm Hg at rest and 180±40 mmHg during exercise, with mean pressure gradient of 80±11.6 (range 40-120) mmHg.

## Results

Neither incidence of paraplegia nor signs of neurological complications or abdominal malperfusion occurred. Mean reduction in systolic blood pressure was 60±25 mmHg without any pressure gradient (p<0.001). During a mean follow-up period of 18.3±3.7 years, there were no reoperations or graft complications. There was no occurrence of pseudoaneurysm formation on the anastomotic sites. Seven (11.6%) had mild/moderate hypertension and are on anti-hypertensive medications. Late mortality is 8.3%.

## Conclusions

This series demonstrates that extraanatomic bypass is safe and achieves satisfactory long-term results in primary or recurrent narrowing of the aortic arch and in patients with severe diffuse and long segments of hypoplasia of the aortic arch.

